# Treatment of chronic HBV hepatitis – between immune control and virological control

**DOI:** 10.1186/1471-2334-14-S7-O10

**Published:** 2014-10-15

**Authors:** Cristina Popescu, Alina Lobodan, Mihaela Rădulescu, Anca Negru, Violeta Molagic, Adriana Hristea, Roxana Petre, Daniela Munteanu, Ruxandra Moroti, Raluca Năstase, Raluca Dulamă, Cătălin Tilişcan, Raluca Mihăilescu, Irina Lăpădat, Ligia Ionescu, Liliana Ion, Victoria Aramă

**Affiliations:** 1 National Institute for Infectious Diseases "Prof. Dr. Matei Balş", Bucharest, Romania; 2 Carol Davila University of Medicine and Pharmacy, Bucharest, Romania

## Background

According to international guidelines, the treatment of HBV hepatitis can use both pegylated interferon (IFN) and nucleoside/nucleotide analogues with high genetic barrier (NNA). The main advantage of IFN based regimen is the possibility of immune control after a therapy with finite duration. The main advantage of NNA is the virological control during lifelong therapy. Objectives: To estimate the level of immune control after IFN therapy and the level of virological control during NNA.

## Methods

Retrospective analysis of HBV infected patients treated in Third Department of Matei Bals Institute, between 2008 and 2014: group 1 – patients who finished IFN therapy and group 2 – patients who received more than 6 months of NNA.

## Results

Of more than 1500 HBV infected patients monitored in our Department, 213 patients received antiviral therapy: 64 patients IFN and 149 patients, NNA. Fifty-six patients in group 1 and 129 patients from group 2 met the inclusion criteria. The demographic characteristics were: in group 1 mean age – 38.51year-old and sex ratio M:F = 1.4:1 and in group 2 mean age – 47.32 and sex ratio M:F=2.2:1. The rate of immune control (defined as HBV viral load <2000 IU/mL) in group 1 was 41%, the mean duration of follow-up was 41.21 months. Thirty-two patients from group 1 had a viral load >2000 IU/mL during the follow-up period and were subsequently treated with NNA after a mean period of 14.15 months. HBsAg loss was obtained in 6 patients (10.71%) and anti-HBs seroconversion in 3 patients (23.21%). Thirteen patients were HBeAg positive and 5 of them developed anti-HBe antibodies (38.46%). Other 2 patients had HBsAg negative at EOT but after 6 months, HBsAg was positive again. In group 2 the rate of virological control (defined as undetectable viral load) was 77.34%. In 3 cases virological failure was recorded. Of 29 patients without virological control, 24 had viral load >LLQ. The mean duration of NNA therapy was 32 months. Nineteen patients had HBeAg positive and in 5 cases (26.31%) anti-HBe seroconversion was obtained, after a mean period of 36.4 months. The mean duration until viral load was undetectable was 32 months. Only one patient registered HBsAg loss, without anti-HBs seroconversion (0.77%).

## Conclusion

After IFN therapy more than 40% patients obtained immune control. This rate could be higher if response guided therapy is used. During NNA therapy the rate of virological control is almost 80% but lifelong therapy is necessary.

